# Interpreting expressive performance through listener judgments of musical tension

**DOI:** 10.3389/fpsyg.2013.00998

**Published:** 2013-12-30

**Authors:** Morwaread M. Farbood, Finn Upham

**Affiliations:** Department of Music and Performing Arts Professions, New York UniversityNew York, NY, USA

**Keywords:** tension, continuous response analysis, activity analysis, expressive performance, trend salience, harmony

## Abstract

This study examines listener judgments of musical tension for a recording of a Schubert song and its harmonic reduction. Continuous tension ratings collected in an experiment and quantitative descriptions of the piece's musical features, include dynamics, pitch height, harmony, onset frequency, and tempo, were analyzed from two different angles. In the first part of the analysis, the different processing timescales for disparate features contributing to tension were explored through the optimization of a predictive tension model. The results revealed the optimal time windows for harmony were considerably longer (~22 s) than for any other feature (~1–4 s). In the second part of the analysis, tension ratings for the individual verses of the song and its harmonic reduction were examined and compared. The results showed that although the average tension ratings between verses were very similar, differences in how and when participants reported tension changes highlighted performance decisions made in the interpretation of the score, ambiguity in tension implications of the music, and the potential importance of contrast between verses and phrases. Analysis of the tension ratings for the harmonic reduction also provided a new perspective for better understanding how complex musical features inform listener tension judgments.

## Introduction

The percept of musical tension provides a window into the disparate components that comprise an expressive performance. Performers' interpretations of a composed piece highlight structural features of the music, shaping the listener's perception of both apparent and unusual aspects of the score (Palmer, 1996). In addition to explicit tempo and dynamics markings, the intrinsic structural features of the score—the harmony, melody, and hierarchical grouping structures—feed into and are reinforced by the expressive interpretation of the performer. Musical tension is a function of both the structural features inherent in the score and the expressive components contributed by the performer.

Tension has long been central topic of interest in music theory (Lerdahl and Krumhansl, 2007), and since the 1980s, it has also been the focus of numerous empirical studies. These studies have examined various aspects of musical tension. However, most of them either focus on specific features or do not attempt to explain how disparate features interact and integrate from a global perspective. The current study offers an explanatory model for how listeners perceive global tension that builds and improves upon an earlier cognitive, computational model (Farbood, 2012) alongside a detailed analysis of tension-rating differences between multiple interpretations of the same musical material. The musical and auditory features that are incorporated into this model include loudness (dynamics), tempo, onset frequency of note events, harmonic tension, and melodic pitch height.

One of the most frequently discussed features with respect to tension is loudness. There have been a number of studies that have identified loudness as a significant contributing factor to tension (Nielsen, 1983, 1987; Krumhansl, 1996; Ilie and Thompson, 2006; Granot and Eitan, 2011). Another commonly discussed feature is tempo, particularly with respect to the effect of rhythm and timing on tension (Krumhansl, 1996; Ilie and Thompson, 2006). Despite the observed contributions of tempo, the effect of rubato on tension perception (as defined by highly local changes in tempo) is unclear (Fredrickson and Johnson, 1996). These local changes might be better quantified in terms of onset frequency of note events, which has also been observed as a textural feature contributing to tension (Farbood, 2012).

A significant number of studies have examined harmonic tension (Nielsen, 1983, 1987; Bigand et al., 1996; Krumhansl, 1996; Bigand and Parncutt, 1999; Toiviainen and Krumhansl, 2003; Lerdahl and Krumhansl, 2007). Most of these studies have explored the psychological validity of Lerdahl's tonal tension model (1996; 2001), and the results have generally indicated that the model accurately predicts harmonic tension. Furthermore, the hierarchical component of the model—a reflection of the tonal context of a given chord—is an essential element for quantifying harmonic tension. Lerdahl's model also has a melodic “attraction” component; however, this aspect of the model has not been supported by empirical evidence. It appears that melodic contour, in terms of pitch height, contributes to global tension as a factor distinct from harmonic or tonal context (Nielsen, 1983, 1987; Bigand et al., 1996; Krumhansl, 1996; Granot and Eitan, 2011; Farbood, 2012).

Tension has also been linked to expectation by music theorists. Margulis's model of melodic expectation (2005), which combines elements of Narmour's (1990, 1992) implication–realization model of melodic expectation and Lerdahl's (2001) tonal pitch space and melodic attraction models, outlines three possible tension responses that listeners may experience: surprise-tension, which correlates inversely with expectancy ratings (i.e., something that is predictable generates little tension), denial-tension, the result of the difference between what is most expected and what actually occurs, and expectancy tension, which is related to the strength of the expectancy that has been generated about future events. Huron (2006) proposes a more general model of expectation that has a tension component related to arousal, corresponding to the physiological response generated when a listener is preparing for an upcoming event.

In addition to Huron's work, there have been other researchers who have suggested links between tension and affective arousal (Krumhansl, 1997). In some empirical work, it appears researchers assume that overlaps between tension and arousal exist, or they use terms that seem equate the two concepts (Rozin et al., 2004; Eerola and Vuoskoski, 2010; Olsen et al., 2010; Lehne et al., 2013). However, this connection has not been explicitly addressed or explored anywhere. The term “tension” has been, in general, used rather broadly in an under-defined manner; for further discussion about this, as well as a more extensive review of the tension literature, see Farbood (2012).

Tension as a measure is particularly useful from an empirical perspective due to the fact that listeners evaluate it with consistency, as indicated in previous studies by high within-subject and between-subject agreement for discrete and continuous tension judgments (Bigand et al., 1996; Farbood, 2012; Upham and Farbood, 2013). Average continuous tension judgments also appear not to be influenced by the musical preferences of listeners (Lychner, 1998) or to change with familiarity to musical stimuli (Fredrickson, 1999).

The reliability of tension ratings and its function as an emergent phenomenon arising from the interaction and integration of multiple, disparate musical parameters make it an effective high-level abstraction with which to examine the psychology of expressive performance and listener interpretation. However, the exploration of continuous tension ratings, in particular the examination of differences in ratings between related musical works or excerpts, has been severely limited by the methodological challenges of time-series analysis. New methods, in particular Activity Analysis (Upham and McAdams, unpublished manuscript), make it possible to investigate these responses in greater temporal detail than has previously been statistically defensible, providing new insights into the time course of tension ratings and agreement between responses.

In this paper, we focus on two previously unexplored aspects of musical tension: (1) the individual timescales at which disparate parameters contributing to tension are processed, and (2) detailed differences in how participants rate similar excerpts. We examine tension from two very different angles—from the perspective of the average tension response and from the ratings identifying tension-related differences between the stimuli. On both accounts, novel approaches to analyzing continuous tension data are explored, providing new insight into how tension is perceived and processed. From a broader cognitive point of view, timescales for feature processing can be viewed from a “levels-of-processing” perspective (Craik and Lockhart, 1972; Craik, 2002), where depth of memory-encoding operations are related to retrieval times. Through our modeling approach, we test the hypothesis that musical features requiring higher levels of cognitive processing, such as harmony, contribute to tension on longer timescales than low-level auditory features such as loudness and onset frequency.

## Materials and methods

### Participants

A total of 29 subjects took part in the experiment, of which four were excluded from this analysis. The remaining 25 participants were primarily undergraduate and graduate students at New York University, mean age 25.16 years (*SD* = 6.50), 11 female, 14 male. Subjects had an average of 9.78 years of formal training on a primary musical instrument (*SD* = 5.18) and an average self-rank in instrumental skill level of 3.90 (*SD* = 0.94) on a scale of 1–5. Average number of semesters of college-level music theory training was 3.32 (*SD* = 2.43) and the mean overall self-ranked musical training level was 3.52 (*SD* = 0.92), where 0 = no training and 5 = professional-level training. The four outlier participants were excluded for not completing the tension-rating task. In the first case, the participant became bored and started hitting random keys, resulting in the data collection interface exiting prematurely; in the other cases, the subjects were unresponsive and did not indicate any tension changes during some or all presented stimuli.

### Stimuli

**Figure 1:**
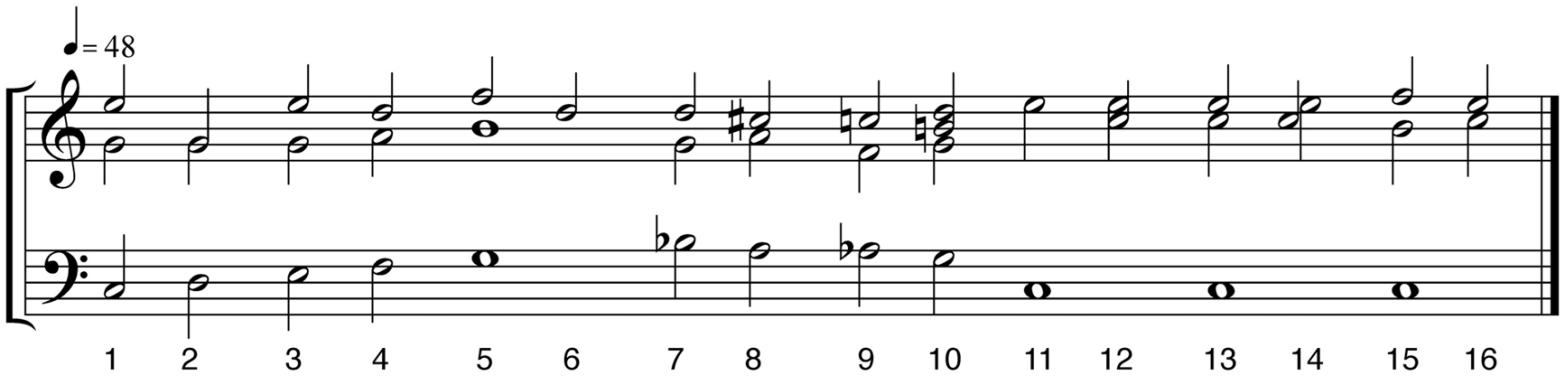
**Harmonic reduction of Schubert's Morgengruss by Lerdahl (2013)**. Chord numbers (not measure numbers) are indicated below the staves.

The stimuli for the experiment consisted of six musical excerpts, two of which are the focus of this paper. Although the task was the same throughout the experimental session, responses to the other four stimuli were collected for a different purpose (as a follow-up to an fMRI study). These other stimuli were an original 4′15″ excerpt from a Brahms piano concerto and three scrambled versions of it. The two stimuli used for the current study are a recording of “Morgengruss” from Schubert's *Die schöne Müllerin* performed by Peter Pears, tenor, and Benjamin Britten, piano, and a harmonic reduction of the piece by Fred Lerdahl (Figure 1). The recording, which includes a piano introduction and four repeated verses, has a duration of 3′55″. The harmonic reduction consists of the chord progression for a single verse and is 40 s long; it was rendered in QuickTime MIDI grand piano timbre for the experiment.

### Procedure

Participants were seated in front of a computer and presented the stimuli over Sennheiser HD 650 headphones in a hemi-anechoic chamber. They were asked to indicate changes in musical tension while listening to the stimuli by moving a horizontal slider on a MATLAB GUI that used Psychtoolbox extensions (Brainard, 1997; Pelli, 1997; Kleiner et al., 2007) for audio playback. The slider position was sampled at 10 Hz. After one practice trial with a one-minute-long musical stimulus that was not part of the test set, each audio excerpt was presented twice in a pseudo-randomized order where no stimulus was repeated before all stimuli had been presented at least once, and no stimulus was presented twice in a row (this avoided the situation where the last stimulus in the first set was the same as the first stimulus in the second set). Repeated presentations were utilized because it has been shown in previous work that within-subject consistency is very high for repeated continuous tension judgments of the same stimuli; habituation does not appear to be a problem in tension-rating tasks even after four repeated presentations of the same stimulus (Farbood, 2012).

### Data preprocessing and analysis methods

#### Activity analysis and rating change coordination

While individual raters may vary in whether or not they report changes in tension and how quickly they report these changes, the temporal dynamics of average tension-rating time series should be fairly representative of future listeners' judgments if the responses show a tendency to change at the same moments in time. In the terminology of activity analysis (Upham, 2011), for a given type of activity event (say an increase in tension ratings), the activity level of a given time frame is the proportion of responses showing indicating the event in question. Ratings-increase activity-level time series, which indicate the frequency of reported increases in tension over successive time frames, and ratings-decrease activity-level time series, which indicate reported decreases, describe whether responses actively agree on changes in tension over time. Figure 6, for example, shows tension-rating-change activity-levels for increases and decreases in each verse and the harmonic reduction of the Schubert excerpt; these time series report the proportion of responses that are active in the half-second following the onset of each 16th note, or 1/12th of the measure.

Like the average, calculation of these summary time series is not sufficient to claim that the resultant pattern is stimulus-driven and robust. Rather than validate the average directly, we test the coordination of tension-rating changes by looking at the distribution of activity levels measured for each stimulus compared to the null hypothesis of independent activity events. If responses do not show coordinated change with each other, we do not have sufficient reason to expect another set of responses to the same stimulus would yield the same temporal dynamics in the average of the activity-level time series. Coordination of rating-change activity can then be evaluated via the distribution of activity levels recorded in successive time frames spanning the piece. A simple test against the null hypothesis of independently timed tension-rating changes compares a model of the activity-level distribution for a collection of uncoordinated responses of equally frequent activity events to that of the experimental data. If the ratings are showing concurrent changes in tension, the experimental activity-level distribution will have more time frames of exceptionally low and relatively high activity levels (around 0.5 for rating data).

Built on this process of evaluating coordination of activity in a collection of time series is the Coordination Score. It is helpful to consider which collections of responses, e.g., responses to different stimuli, show more or less coordinated activity. The coordination score is calculated from the averaged negative log of the *p*-value from goodness-of-fit tests on multiple variants of the time series segmented into frames, with data-driven algorithmic controls on the independent model and distribution testing (Upham and McAdams, unpublished manuscript). The coordination test is evaluated using non-overlapping time frames of size greater than the sampling interval. To reduce the impact of arbitrarily cutting up the time series in even time frames and the risk of splitting apart changes related to the same event, the coordination score is calculated from all distinct slicings of the time series into adjacent non-overlapping time frames of fixed duration. The details of the calculation are accessible via the activity analysis MATLAB toolbox (Upham, 2013). The coordination score is a number from 0 to 16 which estimates the degree of coordination of a given activity event in a collection of stimulus-synchronous responses. Scores of less than 2 are equivalent to *p* > 0.01 for the measured activity-level distributions to occur by chance out of independently changing tension ratings (Upham and McAdams, unpublished manuscript). Very high coordination scores indicate that across the responses, activity is very well coordinated and strongly suggests repeatable summary time series, however, this does not mean that all ratings are in complete agreement on how and when tension changes.

The principle of the coordination score for single-activity events can also be applied to evaluate the independence of different forms of activity in the same collection of responses, for example, both increases and decreases in tension ratings (Upham and McAdams, unpublished manuscript). If a collection is coordinated in their activity, increases and decreases should alternate, but if the responses are independent, both types of activity are likely to happen in the same time frames. The coordination between two types of activity are similarly quantified and scored. If a collection of ratings is coordinated in each direction of tension change but fails to reject independence of increases and decreases, this undermines the robustness of the average time series. Its temporal profile, most often the object of analysis in relating continuous-response data to the time course of the stimulating music, is not likely to be robust since contributions of individual responses cancel out rather than strengthen the common progress of tension.

In the analyses below, activity-level time series are used with average time series to describe the four collections of tension ratings, while the coordination scores, rating change increases, decreases, and alternation between the two, provide arguments for which response collections are employed for modeling and comparisons between verses.

#### Feature quantification

As a first step in the analysis procedure, seven musical and expressive features were quantified for the Morgengruss performance: dynamics, tempo, onset frequency, harmonic tension, and pitch height of the melody, inner voice, and bass line. Although this is not an exhaustive list of features, it was deemed sufficient to account for most of the variation in the tension responses.

The note onsets of the vocal line and accompaniment were determined using marker references in Amadeus Pro (HairerSoft, V 1.5.4) in conjunction with the audio-editing program Audacity (V 2.0.0) for locating precise onset times. With some musical discretion, onsets were generally marked at vowel onsets in consonant lead syllables and piano onsets were also included whenever they did not coincide with the vocal line. Onset frequency was calculated directly from the onset times and was quantified as the difference between the maximum event duration in the performance (3.51 s) and the current note-event duration. Thus the shorter the length of the note event was, the higher the onset frequency. Tempo was determined by using beat onsets. In cases where there wasn't an onset available on a beat, the onset time was linearly interpolated from the positions of the last onset and the next available onset.

**Figure 2:**
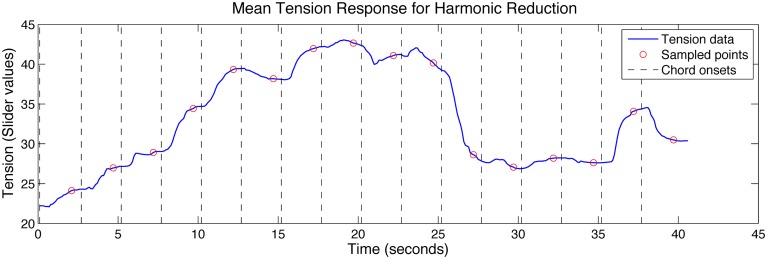
**Mean tension response to the harmonic reduction (second trial only) showing chord onsets and the corresponding sampled points used to describe harmonic tension for each chord change**.

Pitch height of the melody, inner voice, and bass lines were initially encoded as MIDI values and aligned to onset times. The inner voice is only active in the third phrase where the accompaniment echoes the vocal line; however, it was deemed prominent enough to merit addition to the feature set. A harmonic tension graph was created by using the mean tension response to the harmonic reduction stimulus (second rating only; see section Coordination in Tension Ratings for explanation). Tension values sampled 2 s after the onset of each chord were used to quantify harmonic tension for the entire piece (see Figure 2). This sampling lag was employed to compensate for response delay to the event onsets and is in line with lag times proposed by Schubert (2004), who showed that continuous arousal ratings reflect response lags of 1–3 s to musical features other than loudness. Although the harmonic reduction was shortened, it covered all the chord progressions in the piece, including the piano introduction (which is, harmonically, a compressed version of the first phrase of each verse). The harmonic tension graph was created by mapping the sampled tension values to the appropriate onset times corresponding to the performance.

Loudness was evaluated directly from the audio file. The analysis was done using Glasberg and Moore's (2002) psychoacoustic loudness model for time-varying sound available as a function in the Loudness Toolbox for MATLAB (short-term loudness output for omnidirectional sound recording) (Genesis, 2009). The output of the model is quantified in terms of sones, a standard unit of perceived loudness; one sone is equivalent the loudness level of a 1 kHz tone at 40 dB SPL (Stevens, 1936).

**Figure 3:**
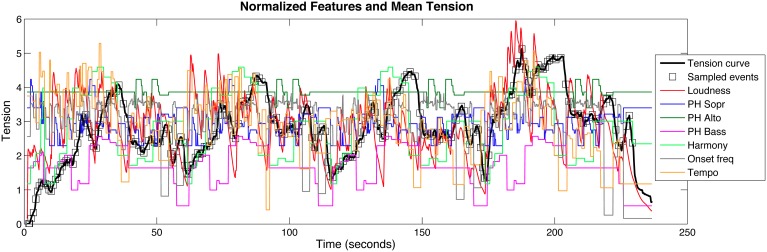
**Mean tension response (second trial only) with sampled points at each beat overlaid with all of the feature descriptions**.

The mean tension response and feature graphs for loudness, tempo, onset frequency, and harmony were then normalized to zero-mean and unit standard deviation (*Z*-score). For pitch height, the mean and standard deviation for all three melodic lines combined were used to normalize the graphs. The normalized mean tension response and features are shown in Figure 3.

#### Parametric tension model

The first analysis approach explored the timescales at which performers and listeners process individual musical features and structures. The vehicle for this analysis was a modified version of Farbood's (2012) trend salience model. The main components of the model consist of (1) an *attentional window* that represents a perceptual moving window in time and extracts a current tension trend; (2) a *memory window*, which immediately precedes the attentional window and represents an abstraction of a previous tension trend; (3) differing weights for each of the musical parameters.

The concept of tension trends is the theoretical core of the model, describing how individual musical and auditory features integrate and interact to produce a global feeling of changing tension. The idea is that the *trend salience* of musical features is the key determiner of tension judgments. The model predicts tension as a function of individual musical features by taking into account the combined directional change of all of the features in the attentional window. This combined directional change, or tension trend, is weighted by what immediately precedes it in the memory window—if the direction of the trend in the memory window matches the current attentional window trend, the magnitude of the cumulative tension trend is additionally increased. These trends are integrated over time to generate a final tension prediction.

The tension trends are essentially the sum of the slopes of all the features for a particular time window. If all features have concurrent negative slopes, the sum of those slopes would indicate a clear decrease in tension for that time window. However, if the slope directions conflict, they might cancel each other out to some degree. The slope of a tension trend at time *t* is defined as

(1) $$s^{\prime }(t)=\beta \sum_{f}w_{f}s_{f}(t),$$

where *s*_*f*_(*t*) is the slope of best linear fit of feature *f* at time *t*; β = 1 if the sign of *s*(*t* − *d*) does not equal the sign of *s*(*t*); β is some positive value, empirically determined, if the sign of *s*(*t* − *d*), where *d* represents the duration of the memory window, is the same as the sign of *s*(*t*); *w*_*f*_ is the weight of feature *f* with ∑_*f*_ *w*_*f*_ = 1. See Farbood (2012) for a discussion on the ideal value of β. The optimal value obtained in that prior study (β = 5) was used in the current analysis. The β component describes the relationship between the memory and attentional windows and adds a non-linearity to the model. The model becomes linear only when the memory window duration is set to 0 s.

Moment-to-moment integration of tension trends are simulated by overlapping moving windows; in this case, the increment is 250 ms, a step-size deemed sufficient for sub-beat time resolution. Each attentional window trend is merged with overlapping previous trends, resulting in recent windows weighted more strongly to simulate memory decay. This result in the slope of the tension curve at time *t* is defined as

(2) $$S(t)=\sum_{\tau \;=\;0}^{\frac{d}{h}-1}s^{\prime }(t-\tau h)k_{\tau },$$

where *h* is the step size of the moving window increment, *d* is the attentional window duration, and *k* is a decay constant for a moving average filter with ∑_*i*_ *k*_*i*_ = 1. From this equation, a final tension value *F*_ten_ at time *t* can be derived:

(3) $$F_{\text{ten}}(t)=h\sum_{i\;=\;0}^{T\;-\;1}S(i)$$

where $T=\left\lfloor \frac{t}{h}\right\rfloor$, and (in this simplified case) *t* is a multiple of *h*.

This model was modified for the current study in one significant respect: instead of employing fixed attentional and memory windows, different time windows were used for each feature in order to optimize the model predictions. Farbood's (2012) evaluation of the model indicated that the ideal values for *d*, in both the attentional and memory window cases, was 3 s. Here, trend salience model predictions were generated *separately* for each feature at different time windows before these individual predictions were integrated into a final tension prediction as described in Equations (2, 3). Furthermore, the feature weights were not used—differences in feature contributions were solely determined by the individual timescales.

#### Alignment

To translate between the recorded stimuli and metrical time, timings for every 1/36th of a measure were interpolated from the onset times in the performance, accommodating quadruple and triple subdivisions of quarter notes. Linear interpolation had to be used in absence of a better model for perceived tempo between onsets, since the resulting inaccuracies would be too small for the timescales employed in the analyses below. This mapping translated participants' tension ratings and musical features, initially sampled at 10 Hz, to metrical time with nearest-neighbor interpolation. Counting measures from the beginning of the verse, the harmonic reduction omitted mm. 7, 18, and 19, since they were repetitions of preceding chords. Matching these ratings to the verses, gaps were either interpolated or left blank, depending on the analysis.

#### Local coordination analysis

While global measures of coordination are useful for confirming shared behavior between ratings of tension to the same stimulus, they are insufficient for comparing ratings to related stimuli with any temporal sensitivity. For that, we employed a nonparametric method for generating distributions of activity levels for each time frame against which actual activity level could be assessed. First, each response time series was reduced to a point process, a sequence of 0 s and 1 s, where 1 indicates the activity in question, such as an increase in tension rating from one sample to the next. Shift values for each response were randomly selected from a time interval (in this case [−5, 5] s), yielding an alternate alignment of these point processes by shifting the complete series in time by the corresponding time value (with the ends looped for continuity), and the activity levels for each time frame were calculated as for the original alignment (Pipa et al., 2008). Repeating this shuffle 1000 times provides a distribution of potential activity levels for each frame for these same responses were they not aligned precisely to the stimulus. This process of shuffling preserves the complex characteristics of these responses, including serial behaviors and longer-term temporal structure (Pipa et al., 2008), while providing a reasonable comparison for assessing the potency of the particular coordination present in the stimulus-aligned response collection. The rank of the experimental activity levels against the alternatives essentially gives a *p* value for that time frame, thus providing a local estimate of likelihood for the experimental activity at each moment. Those moments with activity levels exceeding 95% of the alternatives have been marked as having significant coordination. By this method, it is possible to assess overlapping time frames (non-overlapping time frames are used in the collection coordination score). The basis of this approach is to assume that rating changes are not related to the stimulus—that they are noisy signals rather than clean—and that the points selected are those that have strong evidence to the contrary. This conservative perspective is necessary so long as we do not have a model for forecasting activity levels and the noise in rating changes with more precision. For now, moments of high activity coordination can be considered likely driven by the common stimulus, but moments of less notable activity levels should not be dismissed as noisy and unrelated to the music.

## Results

### Coordination in tension ratings

The coordination of rating-change activity in these responses is significant, allowing for more detailed temporal analysis of the summary reports. Table 1 describes the rating-change activity for each collection of responses (stimulus and presentation order) in terms of average activity rates and rating-change coordination scores. Activity ratings of the second presentation were higher for both increases (Inc) and decreases (Dec). Coordination of rating changes was also higher for the second ratings of the simpler, more sparse harmonic reduction. The combination of higher activity rates and better coordination suggest that the subjects were more confident in their judgments in these later ratings. This is consistent with the hypothesis that subject reports of continuous response are cleaner and faster with increased familiarity with the task and the stimulus, and that agreement between participants for judgment tasks improve with a common context (the full stimulus set), at least for simple stimuli. Tension ratings for the recorded performance manifested very high coordination during the second trial as well, although not necessarily the highest in comparison to the harmonic reduction. With the density of information in the performed stimulus, we should expect that some disagreement in tension ratings will remain despite repetition of the task. In summary, these collections of continuous ratings of tension are strongly coordinated in their rating changes, and we can assume their shared temporal variation to be driven by the common temporal experience of the stimuli, justifying the use of average tension ratings in the modeling analysis described in section Model Optimization and Feature Timescales.

**Table 1:** **Tension-rating change activity and coordination for the first and second presentations of two stimuli, as measured in 1 s time frames**.

**Stimulus**	**Activity rate (Inc)**	**Coordination score (Inc)**	**Activity rate (Dec)**	**Coordination score (Dec)**	**Coordination score (Alt)**
Harmony 1 (40 1 s fr.)	0.21	7.0	0.12	1.8	N/A
Harmony 2 (40 1 s fr.)	0.22	9.9	0.16	7.5	N/A
Performance 1 (235 1 s fr.)	0.17	15	0.15	11	8.9
Performance 2 (235 1 s fr.)	0.19	14	0.17	16	9.7

Tension-rating increases (Inc) are described by their activity rate, the average likelihood of any response reporting an increase in tension during any 1 s time frame, and their coordination score for each response collection. Decreases in ratings (Dec) are described in the same terms, and the last column reports the coordination score for the alternation between increases and decreases in ratings (Alt). Note that the shorter stimulus has no alternating coordination score because it is too brief in duration to evaluate.

### Correlation analysis

#### Correlation of features and mean tension

To get a general idea of how all of the feature descriptions and the mean tension response were related to each other, correlations were performed between all pairs. The time series were sampled at every beat instead of the original 10 Hz rate. Beat sampling was used in all subsequent correlation analyses as well. Spearman's ρ was calculated because the values of several features were not normally distributed. The mean tension response used in all of the following analyses included only the second ratings of the subjects; see section Coordination in Tension Ratings for a detailed explanation for this. All correlations between the mean tension response and features, as well as any other time series in subsequent analyses, incorporate a response lag of 2 s (Schubert, 2004).

The results of the feature correlations, shown in Table 2, generally indicate a weak to moderate positive correlation between features. This includes a weak to moderate correlation between harmony and all features; between dynamics and all other features except pitch height of the melody and inner voice; and between the remaining features (pitch height of the bass, onset frequency, and tempo) and all features except pitch height of the melody. In short, pitch height of the melody was the most negatively correlated with other features. Loudness, pitch height of the bass, harmony, and onset frequency had weak to moderately strong positive correlations with the mean tension response; pitch height of the melody had a weak negative correlation; and all other features had no apparent correlation with mean tension.

**Table 2:** **Spearman ρ values for correlations between features and mean tension (second rating only)**.

	**Loudness**	**Pitch height: melody**	**Pitch height: inner**	**Pitch height: bass**	**Harmony**	**Onset frequency**	**Tempo**	**Tension**
Loudness	–	−0.23	−0.09	0.20	0.30	0.33	0.37	0.52
Pitch height: melody	−0.23	–	−0.15	−0.18	0.19	−0.30	−0.005	−0.17
Pitch height: alto	−0.09	−0.15	–	0.04	0.11	0.26	0.25	0.03
Pitch height: bass	0.20	−0.18	0.04	–	0.34	0.25	0.17	0.43
Harmony	0.30	0.19	0.11	0.34	–	0.06	0.25	0.58
Onset frequency	0.33	−0.30	0.26	0.25	0.06	–	0.33	0.21
Tempo	0.37	−0.005	0.25	0.17	0.25	0.33	–	−0.07
Tension	0.52	−0.17	0.03	0.43	0.58	0.21	−0.07	–

Data points are sampled every beat.

#### Note on reporting of correlations

Correlations are the most common statistic for comparing stimulus features and continuous response data in the existing literature, however, the interpretation of the significance of these calculations has been identified by many researchers as problematic (Schubert, 2002; Upham, 2011, 2012; Alluri et al., 2011). Throughout this paper, we include the correlation values for comparison with numbers published in previous work, but exclude estimates of significance for these calculations since the popular estimation method is inapplicable and no obvious alternatives particularly appropriate for the time series under analysis. The Spearman ρ is still informative for the reader as a relative measure of fit.

### Model optimization and feature timescales

As described above, a trend salience model that predicts tension given a set of continuous feature descriptions was used as a basis for exploring the timescales at which disparate features contribute to global tension. Instead of integrating all features across identical time windows, the original model was altered so that separate attentional and memory window durations were utilized for each feature instead of using fixed weights for each feature. The tension predictions for the individual features at different timescales became the input feature vectors for the global tension prediction. This final integration step did not use a memory window (equivalent to a memory window duration of 0 s) and was integrated in 1 s attentional window frames. The goal was to optimize the output of the model by adjusting the window durations for each feature and correlating the prediction results with the empirical data.

The model was trained on the first half of the data (consisting of the piano introduction and first two verses) and then tested on the final two verses. From a theoretical perspective, it was assumed that the timescales for the three pitch-height descriptions should be identical. That resulted in five memory and attentional window durations to optimize, totaling 10 variables, each with 42 possible durations (0–20 s in 500 ms increments). This high-dimensional space is too large for an exhaustive search, so a step-wise testing procedure was implemented to explore the state space. Starting with all variables at the minimum duration of 0 s, one feature at a time was incremented until the correlation between the model output and mean tension response reached a peak. After optimal values were found for all features, the model output was tested again by exploratory deviations from the fixed optimal values in order to provide further confirmation of the result.

Given this heuristic approach, it is possible that there exists a better solution than what was found—i.e., that the resulting values represent a local maximum. However, the contributions of each feature appear to be predictable around individual maxima for each variable, indicating that a better solution is unlikely. The optimal values found, listed in Table 3, produced a strong correlation with the mean tension response, ρ (Spearman) = 0.86. The results indicate there is a significant difference in the way harmony is processed compared to other features. The memory and attentional windows for harmony were 13 s and 8.5 s, respectively, far longer than any other feature. Dynamics appeared to be the most instantaneously processed feature, having an optimal memory window of 0 s and an attentional window of 2 s. Short windows of ~1 s were optimal for both onset frequency and tempo, while pitch height had slightly longer windows of 2 s.

**Table 3:** **Optimal memory and attentional window durations for each feature**.

	**Features**				
**Window type**	**Loudness (s)**	**Pitch height (s)**	**Harmony (s)**	**Onset frequency (s)**	**Tempo (s)**
Memory	0	2	13	1	1.5
Attentional	2	2	8.5	1	1

**Figure 4:**
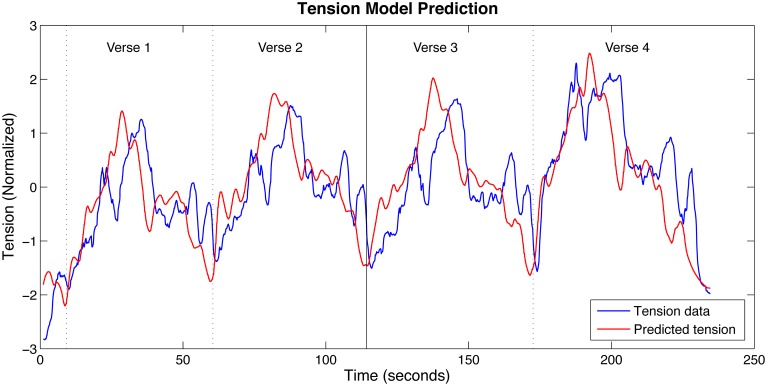
**Trend salience model predictions and the mean tension response**. The solid vertical line indicates the boundary between Verses 2 and 3, the division between the training data (left side) and the test data (right side). The vertical dotted lines mark the other verse boundaries.

These values, optimized only for the training data, were then used to produce a tension prediction for the test data. This also resulted in a strong correlation with the mean tension response, ρ = 0.79, providing some evidence that the model was not overfitting the data. This comes with the caveat that it cannot be determined with certainty that overfitting did not occur since the training and test data are very similar both in terms of the tension responses and feature descriptions (the only substantial difference being the inclusion of the piano introduction in the training data). See Figure 4 for a comparison between the optimized model predictions and the training and test data.

### Comparison between verses and harmonic reduction

The next part of the analysis entailed an in-depth comparison of tension ratings between different presentations of related musical material, namely the individual verses and the harmonic reduction. Given the differences between the stimulus excerpts, we first considered statistics summarizing the cohesion of the collections of tension ratings per excerpt in their second presentations, including rating-change activity coordination. Second, we explored the summary times series for these tension ratings, as aligned to the common musical time course, examining differences between mean tension ratings and moments of extreme coordination. These analyses were primarily descriptive and speculative, demonstrating new approaches to explore and compare continuous ratings of tension. The results provided insight into continuous ratings of tension, potential features to explore in modeling efforts, and interesting examples of musical moments with counterintuitive rating-change behavior.

#### Differences between stimuli

As stimuli, the harmonic reduction and the performed piece are substantially different in character. The harmonic reduction is limited in timbre, with a steady tempo, uniform loudness of notes, very sparse onsets of notes with little impression of meter. Its relationship to the performed verse is restricted to the harmonic sequence, the piano as a sound source, and the approximate duration. As such, it is remarkable how similar the tension ratings were to those of the performed piece, at least as captured by the average.

The verses differ in lyrics, expression, structural order, and other performance parameters. As the participants in the study were US residents, we presume that most if not all were unable to understand the German text. The impact of the lyrics on the differences in tension ratings between verses were therefore likely related to the non-verbal aspects of the musicians' performance. The analysis of tension-rating differences between verses were thus focused on features that were accessible to listeners regardless of language comprehension: articulation, tempo deviation, timbre, and other aspects of affective expression.

#### Summaries of tension ratings per excerpt

**Figure 5:**
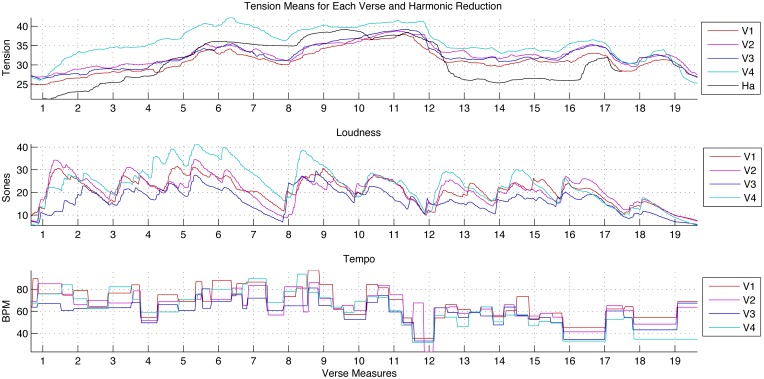
**The average musical-tension-rating time series for the harmonic reduction and each performed verse; the acoustic loudness of each verse in the performed rendition aligned to metrical time; and the onset-determined local tempo, also aligned to the verse measures, for the performed verses**. These last two feature graphs do not include the harmonic reduction, since tempo and loudness were constant across all onsets.

Ignoring the temporal sequence for the moment, Table 4 shows summary descriptions of the ratings for each verse and the harmonic reduction. In terms of absolute tension rating values, the average rating values for each stimulus and excerpt were not very different, ranging from 33 to 37.5 on a scale of 0–100, with the greatest difference being between the harmonic reduction and the fourth verse. Consistent with the idea that ratings of simple stimuli have less opportunity for disagreement, the coordination in rating-change activity was higher for the harmonic reduction, as was the inter-response agreement as captured by the standard deviation ratio. The standard-deviation ratio is a rough measure of agreement between ratings, complementary to the coordination score, and is calculated by dividing the standard deviation of the average rating time series by the average standard deviation of the ratings over time. Values closer to 0 reflect greater noise or contradictory behavior between responses (Upham, 2012). This ratio for the harmonic reduction may, however, be inflated because these ratings include an orienting period, in which participants adjust the slider from the standard initial values programmed in the rating interface to their comfortable rating range during the first 10 s of a rating task (Schubert, 2012). This interval was not a factor for the verses because they were excerpts from the longer rating task consisting of the entire song, which includes an 8 s introduction. Between verses, the coordination scores varied, but they stayed above the significance threshold of 2. The tension ratings ranged more widely (as per the average standard deviation) for the last verse than all previous verses—nearly as much as the case for the harmonic reduction. It is also notable that the activity rates for increases in tension were consistently higher than that of decreases, reflecting the general shape of the average tension time series, with slow increases and quick falls visible in Figure 5.

**Table 4:** **Summary statistics of tension-rating collections per verse and harmonic reduction**.

**Stimulus excerpt (fr.)**	**Activity rate (Inc)**	**Coordination score (Inc)**	**Activity rate (Dec)**	**Coordination score (Dec)**	**Tension mean**	**Tension STD**	**STD ratio**
Harmony (40)	0.22	9.9	0.16	7.5	33.1	8.64	0.72
Verse 1 (52)	0.20	3.3	0.17	6.4	33.8	5.95	0.59
Verse 2 (54)	0.20	5.7	0.16	11	35.8	5.19	0.64
Verse 3 (58)	0.20	2.2	0.16	5.1	35.4	6.17	0.61
Verse 4 (64)	0.19	8.1	0.17	2.9	37.5	7.87	0.71

The collections of tension ratings for each excerpt are described in terms of the average rates of increases and decreases (1 s frames), rating change coordination, average mean tension per response, average standard deviation of tension responses, and the standard-deviation ratio.

#### Comparison of average tension time series between excerpts

Looking at the aligned average tension ratings in Figure 5, the similarity in contour for each verse was notable: rising through the first phrase (mm. 1–7) and cresting at m. 6, rising and staying high for the second phrase (mm. 8–11), falling in the transition to the third phrase (m. 12), and then remaining fairly stable until the small arcs that mark the closing cadence (mm. 16–17) and its repetition (mm. 18–19). At this level, ratings of tension for the harmonic reduction and the verses were quite similar: the tension implications of the harmony and voicing of this reduction were not strongly contradicted by the myriad of other musical features contributing to the perceived tension in the recorded performance.

Across verses, the loudness and tempo of the first phrase (mm. 1–7) were similar or greater than those of the second phrase, mm. 8–11. Despite this, the second phrase always had a higher tension range than the first. The harmonic reduction offers one possible explanation for this pattern, as the tonal character of the second phrase is farther removed from the tonic. Although loudness and tempo have often been noted to have significant influence over the perceived tension and intensity of music, this moment demonstrates that other features can contribute independently.

With regard to absolute tension-rating values, a few peculiarities of the ratings for the harmonic reduction require some explanation. As mentioned before, these values included the beginning of the rating task, the period when participants moved from the initial start position to a stimulus-related value. The relatively steep rise over mm. 1–3, shown in Figure 5, reflected participants' gradual transition from the start position of the rating interface. Another distinguishing moment was the dramatic drop in m. 12. In the harmonic reduction, this measure presents an audible leap downwards of a fifth in the bass line (chords 10–11 in Figure 1). This (relatively) dramatic tonic landing gives the impression of a final cadence followed by a harmonically conservative coda as opposed to the beginning of a new phrase. The strength of the drop in tension at this moment was shared by the first and second ratings of this excerpt, indicating that the perceptual experience was consistent despite exposure to the performed version, which encouraged a different tension profile over the same sequence of chords.

#### Comparison of tension-rating change activity between excerpts

**Figure 6:**
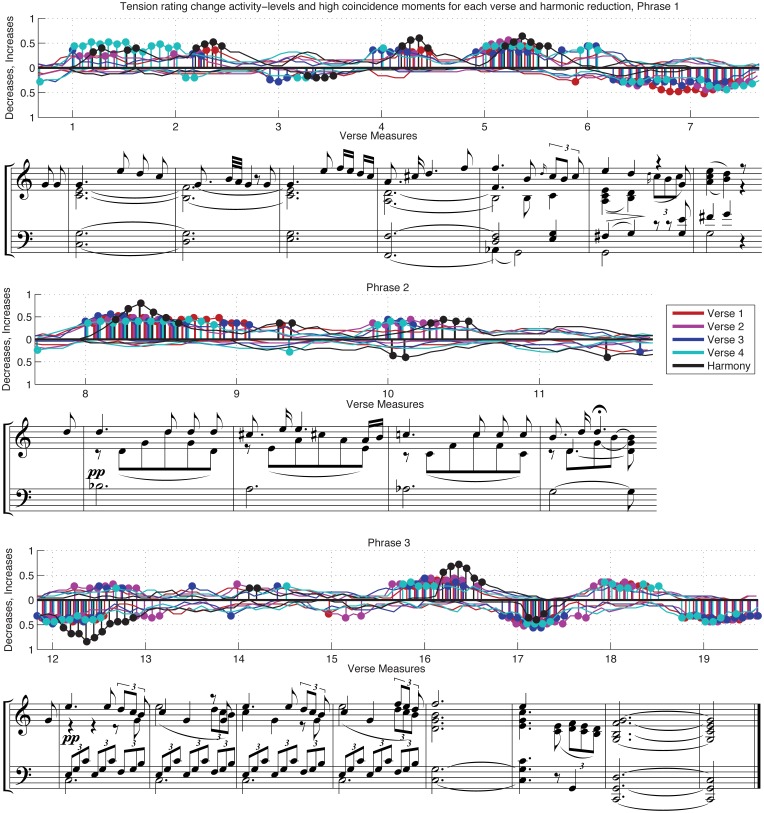
**Rating-change activity-level time series of the harmonic reduction and individual verses**. Bars and dots mark moments with exceptional activity levels (*p* < 0.05, as assessed locally). The notated score includes the vocal line, denoted by the upward stems and broken beaming, with the piano accompaniment on the top staff; the notation format is transcribed exactly as engraved in Schubert (1894) edition.

Additional interesting contrasts can be seen in the rating-change activity levels in Figure 6. Each graph in the figure presents rating-change activity levels for each version over a phrase of music. Each line represents the proportion of responses showing rating increases or decreases in the half-second following that point in the music, calculated for every 16th note onset, or 1/12 of every measure. The lines in the positive range reflect the activity levels for increases in ratings greater than 1% of the rating scale: a line close to zero indicates few reported increases in tension at that point in the music; moments where the line rises above 0.5 in the positive range indicate that over half the participants are reporting tension increases during the corresponding half-second interval. The lines below zero report decreases in ratings; when most participants are reporting decreases in tension, the activity-level line falls below 0.5 in the negative range. Plots of activity-level time series make it easy to see when there is little total rating-change activity (when both lines per stimulus are close to zero), when there are contradictory rating changes between responses, and when ratings alternate between periods of widespread increases and decreases. Figure 6 reports activity over shorter time frames of 0.5 s. The finer resolution is useful for avoiding accidental overlaps of contrary rating changes. However, each frame is unlikely to capture all participants' responses to salient events since response lags vary across participants and contexts. The activity-level time series for each verse and harmonic reduction in Figure 6 are annotated with bars and dots to highlight moments in each excerpt with exceptionally high change activity (*p* < 0.05), as described in section Local Coordination Analysis. The following discussion will concentrate on these “significant” moments as a way to contrast ratings for each verse and the harmonic reduction.

Rating-change activity levels for the harmonic reduction are shown in black in Figure 5. Activity coordination for this excerpt was shaped by the relatively high activity levels in the first half of each measure in response to each new chord or note on the downbeat. Since the time between note events was long (2.5 s), the rating changes may also have been delayed in comparison to naturalistic stimuli because participants did not have precise beat entrainment to help them anticipate the changes at the next downbeat. Nonetheless, these note events provoked widespread changes in tension ratings with little disagreement as to direction of change. The only measure that showed significantly coordinated rating changes in both directions was m. 10 (corresponding to chord 9 in the reduction shown in Figure 1). Around the downbeat, there was a concentration of participants reporting decreases in tension, followed rapidly by a significant number of reported increases. Looking directly at the individual responses, only 10 of 25 subjects indicated increases during the measure, while seven indicated only decreases. Surprisingly, six others—a quarter of the participant group—initially reported decreases in tension in the first moments of the measure before changing directions to indicate an increase 0.5–1 s later; no responses showed the reverse pattern. This change may come from a conflict between participants' expectations and the stimulus, or perhaps these listeners reinterpreted the event after it had sounded. Although the material presented is controlled and sparse, this moment in particular appeared to be controversial and potentially dynamic in the ears of the listeners.

The last verse of the performance had the highest average tension values of all excerpts, as reported in Table 4 and Figure 5. The difference between Verse 4 and the other verses was strongest in the first phrase, with the gain gradually lost over the second phrase. The separation began at the start of m. 1, throughout which increases persisted at significant levels for the full duration of the measure, in contrast to the shorter-lived activity in other verses. Many factors may have contributed to these reported changes in tension. Verse 4 was neither the loudest nor the fastest excerpt, although both of these features were increasing during this interval. Verse 3, the slowest and softest verse, did not yield strikingly lower tension ratings than its predecessors. However, from a qualitative perspective, the beginning of Verse 4 sounds loud and emphatic after such restraint. The sung words at the beginning of Verse 4 come across with great emphasis—the melodic passage is performed for the first time with detached articulation, adding to the contrast. Lastly, structure may also have had an influence; it is possible through these and other cues that listeners were aware that this verse was the last.

Contrasts between repetitions are rarely discussed in models of musical tension, despite their importance in performance practice. Performers generally make contrasts in returning material, particularly when semantic differences such as lyrics support a difference. While the overall tension averages were very similar, there were many other interesting contrasts in rating-change activity between verses that co-occurred with deliberate differences in the performances of the same melodic line and accompaniment.

The second phrase (middle graph, Figure 6) features the harmonic extremes of the piece. This phrase consists of two iterations of a two-measure motive, the second a step down from the first, over new harmonic material. The average tension ratings for all the verses showed a gradual increase in tension that peaked in m. 11. This smooth ascent seems in direct contradiction to the fact that many musical cues would suggest that the second two measures should be lower in tension than the first (in particular the cues encoded for the predictive model). Loudness, tempo, melody, and bass pitch height, even the harmonic tension ratings do not suggest a higher tension level for mm. 10–11. What else in the music could be driving these ratings? Though hard to quantify, this second phrase was performed without release or resolution. Through legato and upward motion in the melodic line, the singer communicated sustained tension—a feeling of not letting go; the last note of the phrase was even extended by a fermata. Though decreases in loudness and tempo often express a move toward calm and relaxation, in this case they might have added to the perceived tension as a signal of active restraint.

The activity levels revealed another layer of complication for interpreting the tension ratings of this second phrase. Although most participants did show a slight increase in tension ratings in mm. 9 and 11, the most concentrated activity occurred in mm. 8 and 10, as can been seen in Figure 6. The “peak” in tension ratings seen in the averages at m. 11 is only the product of diffuse rating changes, including decreases during the fermata, and not a widely shared response to a specific event in the music. It may be that activity levels relate more directly to the feature variations than the average tension ratings, at least for this second phrase.

The specific timing of tension-rating changes to the cadential motion in mm. 16–19 of the performed verses and mm. 16–17 in the harmonic reduction point to the importance of context and clarity of cues. Measures 18–19 in the performed recording were very similar to chords 16–17 in the reduction in terms of harmony, timbre, and voicing. However, the ratings for mm. 18–19 in the performance were never as coherent as those of the harmonic reduction. The ratings for the performed verses anticipated the final event, a tonic arrival, decreasing considerably before its onset. There are two plausible explanations for this: metrical anticipation and contextual richness. As mentioned above, the time between events in the harmonic reduction was too long for participants to predict the exact moment of the next downbeat. This lack of definite pulse would have forced participants to wait for cues, instead of anticipating them. In contrast, the performed version provided listeners with a rich metrical framework. Prepared by temporal expectations, they may have been reporting decreases early in anticipation of a downbeat that had been displaced by the expressive rubato of the performers. Alternately, these decreases may have been the result the stronger cadence in mm. 16–17 preceding this moment, during which participants also indicated tension decreases before the arrival of tonic. Other cues present in mm. 16–17 in the performed version could have encouraged tension-rating decreases before tonic arrival, including dynamic cues in the vocal line, which include timbre and loudness tapering over the last sung syllable of the verse. Like the confusion in m. 10, these moments are reminders that much of perceptual effects of music are not easily deduced from the notated page.

There are many other nuances of performance that could be explored using activity-level time series since they allow for the identification of salient moments of rating change. With more precise representations of tempo variation, articulation, and vocal timbre, it may be possible to quantify the factors behind inter-verse differences in m. 2, the transition to m. 4, the transition to m. 6, and at the end of m. 8. The timing of changes in this collection of responses point to the relevance of context—structural, metrical, and harmonic contrasts—between successive verses and phrases.

## Discussion

This study examined listener judgments of musical tension for a recording of the Schubert song Morgengruss and its harmonic reduction. We focused on two previously unexamined aspects of tension: the differences in processing timescales of disparate expressive and structural features contributing to tension and differences in tension ratings between the verses and the harmonic reduction. The methodologies employed in both set of analyses were novel, providing a fresh perspective on how tension reflects listeners' and performers' musical perceptions.

The first part of the analysis examined timescales of musical feature processing by using a modified version of Farbood's (2012) trend salience model of tension. The model describes tension in terms of a moving attentional window in time that represents a current tension trend. Additionally, a memory window has an increased effect on the magnitude of the perceived trend—whether negative or positive—if the current trend is continuing in the same direction as the previous trend in the memory window. The model was modified by using different memory and attentional window sizes for each feature instead of fixed durations across all features. The goal was to better understand how processing timescales differ between features contributing to tension. This was accomplished by finding the optimal window durations for each feature that resulted in a model prediction best correlated with the mean tension response. The features examined included harmony, pitch height, dynamics, onset frequency, and tempo.

The results indicated that harmony was processed across a far longer time span than all other features, having a combined attentional plus memory window duration of 20.5 s. The feature with the next-longest combined window duration was pitch height at 4 s. Onset frequency, tempo, and dynamics all had combined window durations of ~2 s, however, dynamics had an optimal memory window of 0 s, indicating that loudness induces the most instantaneous tension response. These results align with more general perspectives on working memory such as Craik and Lockhart (1972), who theorized that higher levels of information abstraction are associated with longer persistence in memory. Deutsch and Feroe (1981) suggested that this theoretical framework should apply to music as well.

It is perhaps no surprise that listeners are responsive to highly local changes in loudness. Instinctual response to loudness is a basic, low-level function—anticipating and sensing approaching and retreating objects in the environment is important from an evolutionary perspective; in particular, listeners are highly sensitive to looming sounds (Neuhoff, 1998, 2001; Granot and Eitan, 2011). Tempo perception, predicated on beat induction, requires higher-level abstractions than loudness perception. The optimal combined time windows for tempo in fact encompass a time span (2.5 s) that is just beyond the upper limit for beat induction (2 s; London, 2012). The average tempo for the Morgengruss performance is 60 BPM, meaning that the optimal time windows in this case spanned approximately two and a half beats. The optimal window sizes for onset frequency were slightly shorter than for tempo (a combined duration of 2 s vs. 2.5 s), but they arguably fall under the same general timescale. Onset frequency and tempo are linked; in the simplest case, an increase in tempo of isochronous onsets corresponds directly to an increase in onset frequency.

The optimal window durations for pitch height were approximately twice as long as those for tempo and onset frequency. This reflects the likelihood that melodic contour is processed at a higher level of abstraction than all the other features examined here except harmony. Gestalt perception is a primary factor in melodic contour perception (cf. Meyer, 1956; Tenney and Polansky, 1980; Lerdahl and Jackendoff, 1983; Narmour, 1990), and the results suggest that—at least in the specific case of the Schubert—contour is evaluated over a time window that spans slightly longer than a measure.

The process of tonal induction and harmony perception requires higher-level cognitive abstractions than any other features, and this is evidenced by the long optimal time windows. These findings are in concordance with the result of a preliminary study by Farbood (2010) that examined how the constraints of working memory might affect perception of hierarchical tonal structures in the context of a proposed memory decay component to Lerdahl's (2001) tonal tension model. Farbood analyzed continuous tension responses to a one-minute Bach-Vivaldi excerpt using regression analysis that included harmonic tension, melodic contour, and onset frequency as independent variables. These features were described in terms of change over time spans ranging from 0.25 to 20 s. The results showed that changes in harmony best correlated with the tension data when the time differential was around 10–12 s, while other features best fit the data at a time differential of around 3 s. These results indicated far longer memory effect for harmony compared to other features. It should be noted, however, that harmonic processing is by nature subject to a longer timescale of processing due to the temporal trajectory of harmonic progressions. This potential confound may be a contributing factor in these converging results.

The second part of this study examined the differences in tension ratings for the varying interpretations of the Morgengruss score across the four performed verses and the harmonic reduction. While the average tension ratings for the verses were very similar, differences in how participants reported tension changes support the importance of performance decisions that provide nuance to the interpretation of the score. Performer-controlled features such as loudness and tempo, less-easily-quantified articulation and sustain, and contrasts between successive verses were each highlighted as likely factors for these subtle but substantial differences in the tension ratings.

Analysis of ratings of the reduction underscored the importance of harmony by providing an explanation for the high tension ratings in the second phrase of each verse. It also offered an interesting case study for analyzing activity in tension ratings; having a reduction representing at least one complex feature made it easier to make sense of the complicated information contributing to the dynamics of average tension ratings.

There is some precedent for comparing tension or other continuous ratings between interpretations of a common work (Fredrickson and Johnson, 1996; Goodchild et al., 2010), as well as comparisons between section repetitions within pieces (Livingstone et al., 2012). In the current study, the expressive range and multiple verses in Morgengruss provided particularly fruitful data for exploring the effects of performance parameters. In prior work, most comparisons of performed interpretations of a common score have been limited to noting dramatic contrasts or making vague claims due to insufficient tools for assessing the reliability of differences or the significance of small changes in the average time series. By using rating-change activity levels and a novel approach to assessing the significance of coordination at each moment of the music, it was possible to detect salient differences in tension-rating behavior at a finer temporal scale than employed in previous work. Combining activity analysis with local coordination measures also provided new evidence that contrary rating activity is a common and important phenomenon that justifies using representations of continuous responses that acknowledge the multiplicity of perceptions reported.

While the coordination analyses of the tension-rating changes were data-driven and robust, the comparisons between responses across different stimuli were not performed systematically. Interpretation of contrasts between verses were informed by musical experience and need to be tested, perhaps with other examples of controlled stimuli. The study of Romantic lieder in particular would be greatly improved with the incorporation of a dynamic model of tempo prediction, sensitive to the grouping implications of different degrees of rubato and descriptors of the sung timbre and articulation.

Comparisons of the tension ratings between verses also raises the question of structure and tension judgments, in particular, the importance of contrast between verses. How the same moment in a verse is treated from one iteration to the next warrants more attention since it might be a means to capture the role of form in musical memory and the continuous experience of music. What would it mean for a model to include information about the previous presentation of the same material? While the idea of contrast between repetitions affecting tension is intuitive and familiar to performers, testing this hypothesis would require more instances of specially composed stimuli.

The effectiveness of any analysis of tension is dependent on the inclusion of a complete set of features contributing to tension. Although we examined several key parameters, they do not represent an exhaustive set of features that account for all tension variations perceived by the performers and listeners of the Schubert. Perhaps the most significant feature that requires future examination is timbre. Defining the perceptual dimensions of timbre is a difficult task, and that is perhaps one reason why timbre is underexplored in the tension literature. Prior studies that have explored timbral tension in some capacity have looked at features such as roughness, brightness, spectral flatness, and density (von Helmholtz, 1877; Plomp and Levelt, 1965; Hutchinson and Knopoff, 1978; Nielsen, 1987; Krumhansl, 1996; Pressnitzer et al., 2000; Dean and Bailes, 2010). Further work needs to be done to empirically investigate and confirm the primary components that contribute to timbral tension. Given this knowledge, it might be possible to better understand the nuances in a solo vocal performance such as the Schubert, where tone and articulation are of great expressive importance.

Although additional experiments using more varied musical stimuli would undoubtedly strengthen and solidify these findings, the analyses and observations made in this study present new perspectives on tension. They help illuminate the complex process of how tension is affected by performance decisions and how listeners respond to those differences. Having a deeper understanding of tension perception can help us better grasp the interplay between expressive performance, listener interpretation, and musical structure.

### Conflict of interest statement

The authors declare that the research was conducted in the absence of any commercial or financial relationships that could be construed as a potential conflict of interest.
